# *Zingiber officinale* Rosc. in the Treatment of Metabolic Syndrome Disorders—A Review of In Vivo Studies

**DOI:** 10.3390/ijms232415545

**Published:** 2022-12-08

**Authors:** Ewelina Gumbarewicz, Agata Jarząb, Andrzej Stepulak, Wirginia Kukula-Koch

**Affiliations:** 1 Department of Biochemistry and Molecular Biology, Medical University of Lublin, Chodzki 1, 20-093 Lublin, Poland; 2 Department of Pharmacognosy with Medicinal Plants Garden, Medical University of Lublin, Chodzki 1, 20-093 Lublin, Poland

**Keywords:** ginger, inflammation, obesity, cardiovascular system, molecular biology, Zingiberaceae

## Abstract

Inflammation is a protective reaction of the innate immune system as a response to imbalances caused by a specific stimulus, a disease or a pathogen. A prolonged inflammatory condition may lead to the development of metabolic syndrome, which affects more than one-fourth of the world’s population. This condition leads to the development of multi-organ disorders based on disrupted blood lipid and sugar levels, hypertension and oxidative stress. The review aims to present *Zingiber officinale* Rosc. as a plant that exhibits a variety of healing properties and restores the organism’s equilibrium. Ginger (GI) rhizomes have been commonly used in traditional medicine to treat arthritis, stomach ache, nonalcoholic fatty liver disease, rheumatism, nervous system syndromes, asthma, diabetes and nausea caused by pregnancy or chemotherapy. This review gathers together data from in vivo experiments related to the application of ginger for the treatment of inflammatory conditions, obesity, diabetes and other related disorders as a consequence of metabolic syndrome, including the confirmed molecular mechanisms of action.

## 1. Introduction

In recent years, particular attention has been paid to natural substances that are present in the environment. Extracts from various parts of plants have been tested to identify cures for different diseases lowering the quality of life or even causing patient death. Some of the most common ailments are related to heart diseases and obesity, which often accompanies the former. These disorders have been associated with the development of inflammation, dyslipidemia, hypertension, cardiovascular disease and diabetes [1]. These abnormalities are known as metabolic syndrome (MeS), which is now regarded as a pandemic because it affects one-quarter of the world’s population [2].

Zingiberaceae is a botanical family that contains over one thousand three hundred species of plants. These monocotyledonous perennials cover the areas of Southeast Asia but also tropical parts of Australia and South America. Ginger (*Zingiber officinale* Roscoe)—the most well-known and widely spread representative of this botanical family—is used as a spice all over the world, not only because of its characteristic aroma and pungency but also due to the medicative potential of its rhizomes [3]. The plant has been used over the ages in traditional Chinese medicine and the Indian ayurvedic system of medicine. Ginger deserves special attention due to its therapeutic applications. Powdered rhizomes and extracts from the plant are used to treat arthritis, stomach ache, nonalcoholic fatty liver disease, rheumatism, nervous system syndromes, asthma, diabetes and nausea caused by pregnancy or chemotherapy [4,5].

**Figure 1:**
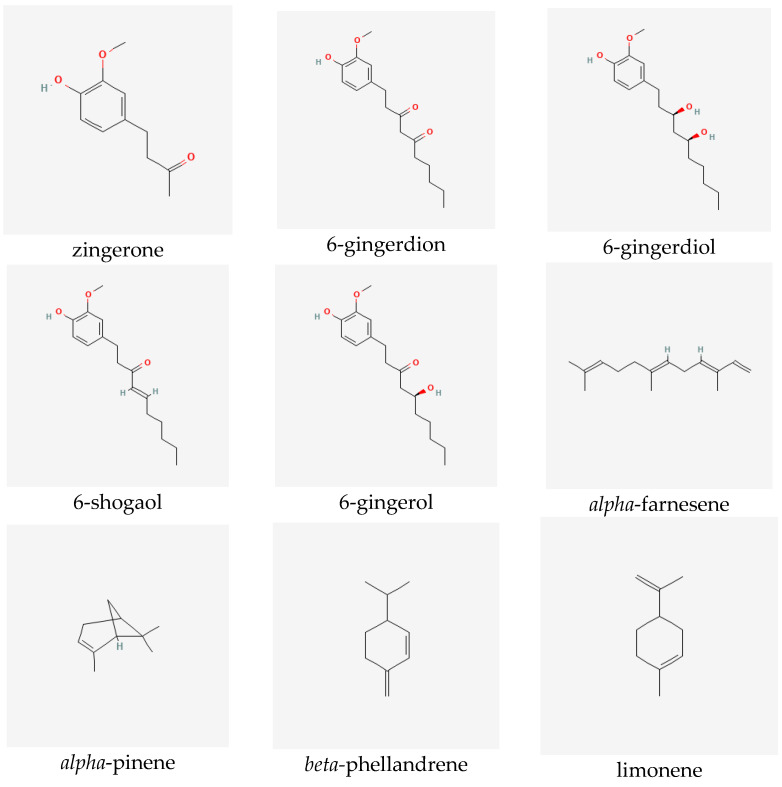

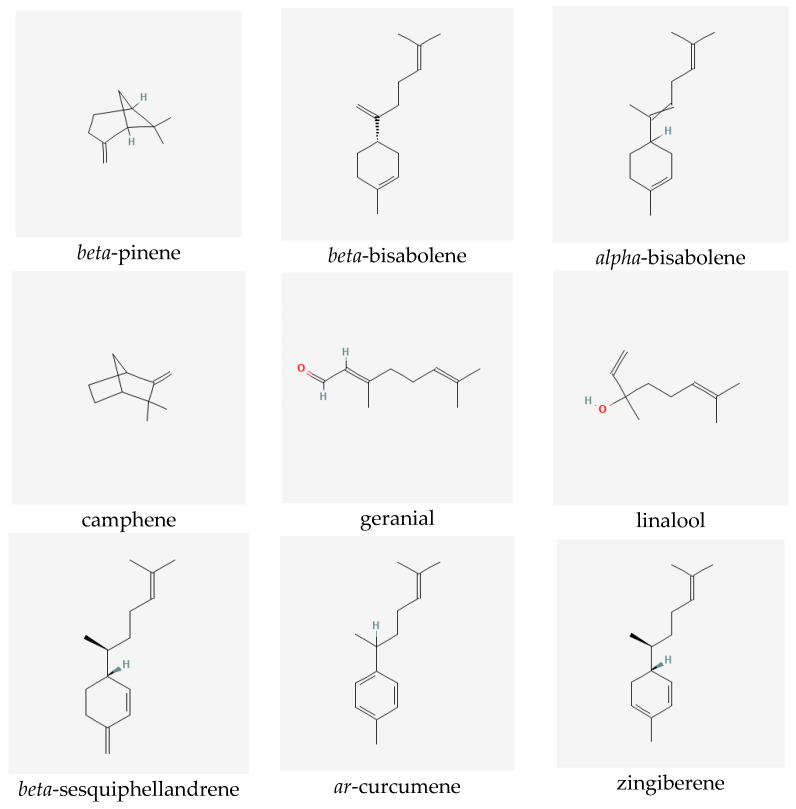
Selected secondary metabolites identified in ginger rhizomes.

Ginger extracts are characterized by the presence of two groups of metabolites of pharmacological significance: non-volatile phenolic components and volatile constituents. Terpenes form approximately half to more than three percent of all compounds present in the rhizome. However, their composition in the plant may vary depending on the origin of the ginger [6]. The characteristic and specific aroma of ginger, regardless of the place of its occurrence and type, is mainly caused by sesquiterpene hydrocarbons, such as zingiberene (constituting about fifty percent of all the components) [7]), *ar*-curcumene, *beta*-bisabolene, *alpha*-farnesene and *beta*-sesquiphellandrene, as well as monoterpene hydrocarbons, such as citral (*cis* and *trans* forms), linalool, geranial, limonene, camphene, *alpha*- and *beta*-pinene and *alpha-* and *beta*-phellandrene [6,7,8]. Non-volatile components, which constitute five to eight percent of the oleoresin obtained from ginger rhizomes, consist of phenolic compounds, namely phenylalkanes, called gingerols, the most important of which is 6-gingerol, which is responsible for the sharp taste of the plant [9]. Other substances with a similar pungent taste are present in smaller quantities in the extracts. They include zingerone, gingerdions, gingerdioles and shogaols, of which the dehydrated derivatives of the former are formed at high temperatures or during the storage of the plant (see Figure 1) [10].

As mentioned above, ginger is a plant with multidirectional actions and healing properties. Therefore, it is constantly under investigation with respect to its further applications as a plant-derived drug and its molecular mechanisms of action. Therefore, the aim of this review is to create a comprehensive overview of the anti-inflammatory potential of ginger extract and its single components that explain its administration in the treatment of diseases affecting the majority of civilizations. These properties are precious, as the majority of them progress in cases of impaired inflammatory regulation. The data presented in the review are based on both animal and human studies from recent years [11].

## 2. Methods

The research data collected for this review manuscript were obtained from the following scientific databases: Scopus, ScienceDirect and Pubmed. The articles that are listed in the review appeared in the search result list after introducing the following keywords: “ginger”, “inflammation”, “Zingiber officinale”, “gingerol”, “shogaol”, “anti-inflammatory”, “anti-inflammatory”, “ginger rhizomes”, “ginger components”, “natural products ginger”, “in vivo”.

## 3. Pharmacological Properties of Ginger

**Figure 2:**
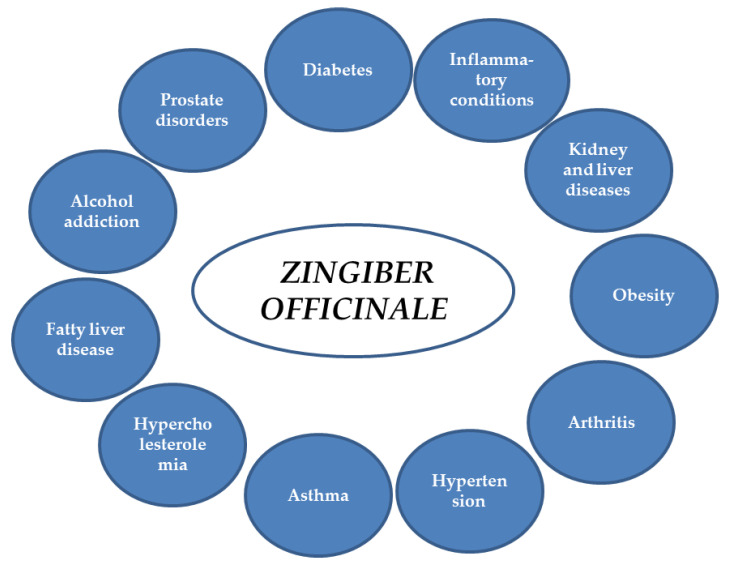
Potential applications of ginger.

**Figure 3:**
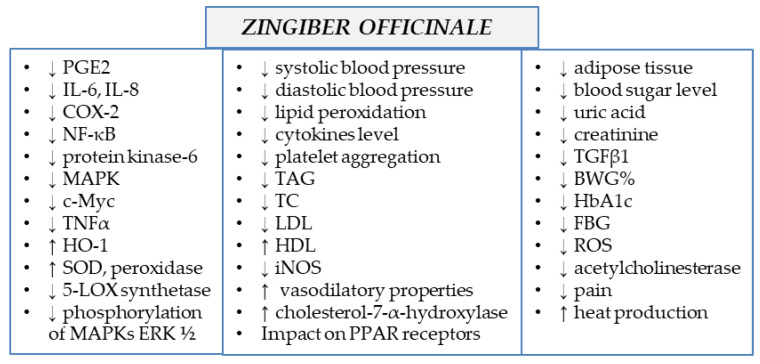
Molecular mechanism of action of *Zingiber officinale* rhizomes (↓—downregulation/decrease, ↑—upregulation/increase).

Ginger extracts and single components isolated from the plant matrix were proven to exhibit anti-inflammatory properties. Due to this potential, the plant is considered to be useful for the treatment of various diseases whose onset includes inflammatory conditions. Below, the detailed characteristics of ginger and its properties are listed, together with the proven molecular mechanism of action (see also Table 1 and Figure 2 and Figure 3).

### 3.1. Anti-Inflammatory Properties of Zingiber Officinale

The majority of diseases, including the chronic ones, are related to the inflammatory state of the organism. Anti-inflammatory drugs—despite their efficacy—can cause various side effects. They also require high doses in order to remain efficient, which results in problems when delivering them orally and intravenously over a long period of time [12,13]. This is why there are clinical trials aiming to introduce natural compounds for treatment of inflammation, whether alone or in combined therapy with routinely used anti-inflammatory drugs. Some of the trials introduce specific nanoparticles with active components [14,15] to improve the method of drug delivery. Interestingly, some of the novel drug delivery forms include preparations with ginger. Nanoparticles loaded with the ginger constituent 6-shogaol soothed ulcerative colitis symptoms and aided in colitis wound repair [16]. At the same time, intensive pre-clinical studies are ongoing in order to introduce ginger-related compounds into clinical settings.

The molecular targets of ginger and its constituents have been evaluated in both in vitro and in vivo assays.

In vitro studies revealed that ginger alcoholic extract (up to 100 µg/mL), 6-gingerol and 6-shogaol (both up to 1 µg/mL) inhibited pro-inflammatory markers such as PGE2, IL-6 and IL-8 on the protein level and decreased the mRNA expression of iNOS and COX-2 in a colon adenocarcinoma Caco-2 cell line [17].

It has also been demonstrated that 6-shogaol–one prevents the development of inflammation and reduces oxidative stress through Nrf2 signaling in human epidermal keratinocytes (HaCaT cells) in vitro. Additionally, 6-shogaol–one increases the concentration of glutathione and activity of NAD(P)H: quinone oxidoreductase (NQO1) [18]. Other studies [19,20,21] showed that [6]-gingerol attenuates the induction of pro-inflammatory signal transduction, including the NF-kB, protein kinase-C and mitogen-activated protein kinases (MAPK) pathways in HaCaT cells.

The anti-inflammatory activity of ginger and its metabolites, revealed by in vitro assays, were supported and confirmed by in vivo studies. Mice with dextran-sulfate-sodium-induced (DSS-induced mice) colitis treated with ginger extract (50% ethanol) administrated orally for 21 days at increasing concentrations of up to 500 mg/ kg b.w. displayed a reduced number of neutrophils and reduced concentration of pro-inflammatory cytokines IL-6 and TNF-α at all the tested doses of the extract in the examined tissue. Additionally, ginger extract suppressed the mRNA expression of IL-1β, as well as IL-6 and TNF-α, in a dose-dependent manner in DDs-induced mice [22]. Finally, ginger extract enhanced the mRNA expression of tight junction proteins (ZO-1, occludin, E-cadherin, mucin-1, mucin-2) compared to control group of mice [22]. Noteworthy is the fact that ginger facilitated the decrease of body weight and increased the disease activity index (DAI) as well.

Similar changes, including a decrease in the inflammatory cytokine concentration (IL-1β, IL-6 and TNF-α) and a decrease in the neutrophil levels after the oral administration of 6-gingerol were observed in an ischemia–reperfusion (I/R) rat model. The in vivo studies performed by Li and co-investigators on I/R-induced male Sprague-Dawley rats fed once a day for three consecutive days before the application of 6-gingerol suspended in water through oral gavage (25 and 50 mg/kg b.w.) demonstrated a decrease in the neutrophil levels and inflammatory cytokines IL-1β, IL-6 and TNF-α [21], showing that ginger compounds can exert their effects in a wide variety of pathological conditions associated with inflammation. It seems that the anti-inflammatory activity of ginger compounds can be reinforced by additional factors. The prolonged simultaneous administration of ginger extract to rats (50 mg/kg b.w.) together with *Lactobacillus acidophilus* (10^9^ CFU) showed the deepened inhibition of oxidative stress vs. the single treatments. In more detail, these in vivo studies revealed that this mixture significantly lowered the malonyl dialdehyde (MDA) levels and increased both catalase (CAT) and sodium dismutase (SOD) activity, the enzymes responsible for reactive oxygen species (ROS) elimination. Moreover, it was demonstrated that the ginger extract/Lactobacillus acidophilus composition reduced the TNF-α concentration in the serum of the treated rats, as well as the downregulation of mRNA of genes involved in inflammatory pathways, including COX-2, iNOS and c-Myc [23].

Other studies also testify to the anti-inflammatory properties of ginger essential oils (GEO). GEO administered at the concentrations of 200 and 300 mg/kg to ginger-treated experimental autoimmune encephalomyelitis (EAE) mice was observed to decrease the expression of IL-12 and TGF-β in the central nervous system and serum [24].

GEO was also tested as a possible nephroprotective agent in a rat nephrotoxicity model induced by the administration of 1.0 mg/kg i.p. of cadmium (Cd) that was characterized by elevated blood levels of urea, creatinine and blood urea nitrogen (BUN). After the treatment with ginger essential oil (50 mg/kg b.w., p.o.), it was found that the levels of these biomarkers decreased. The possible mechanism can be correlated with changes in the pro-inflammatory cytokine levels, which rise after Cd administration. Moreover, Cd activates renal adenosine deaminase activity (ADA), which is related to the purinergic response to several kidney disorders. A similar effect concerning the activity of adenosine deaminase was observed in the hippocampus and pre-frontal cortex of Cd-treated rats after ginger and turmeric supplementation. This action has the neuroprotective effect of increasing the adenosine levels during inflammation. Ginger administration protects the adenosine levels and offers renoprotection during renal failure after the actions of toxic substances [25]. Cd administration also affects other tissues, such as nerve tissues. Recent studies showed that Cd reduced the IL-10 levels but increased the IL-6 and TNF-α levels [26]. Moreover, Cd can induce neuroinflammatory events through blood–brain barrier leakage, microglia activation and the infiltration of the brain by immune cells [17]. The simultaneous usage of ginger oils and turmeric rhizomes showed an inhibitory effect relative to the pro-inflammatory cytokine levels. Additionally, the activity of acetylcholinesterase, elevated levels of which are observed in inflammation, was inhibited by the co-treatment of ginger oils with turmeric oil in the hippocampus and pre-frontal cortex. This may be one of the suggested mechanisms for the treatment of Alzheimer’s disease (AD) [26].

Another factor that causes damage to the kidney tissue is γ-Ray exposure during radiotherapy treatment. Ginger extract was given to rats before radiation exposure. It was found that the levels of pro-inflammatory enzymes such as ODO, iNOS, COX-2 and pro-inflammatory cytokines (TNF-α, IL-1β) were decreased. Additionally, ginger suppressed the activity of 5-lipooxygenase synthetase and iNOS activity. At the molecular level, ginger inhibited the phosphorylation of MAPKs and ERK1/2 and the activation of NF-kB [27].

Based on the anti-inflammatory properties of ginger, which are related to the decrease in the pro-inflammatory levels of cytokines, prostaglandins and leukotriens, it has been reported that ginger can suppress the side effects after the administration of morphine. Ginger acts by using vanilloid receptors [28]. The long-term usage of this opioid drug activates the astrocytes and microglia, which are responsible for releasing inflammatory cytokines and ROS, leading to the development of neuroinflammation in the nerve tissue. On the molecular level, it was stated that the p38 MAPK pathway contributes to the overproduction of these cytokines, and it was found that ginger suppressed this signaling pathway during in vivo experiments [29].

The anti-inflammatory properties of ginger support its application in the treatment of arthritis. Ginger preparations modify the onset of osteoarthritis, and they have protective and regenerating effects on articular cartilage. Due to the fewer side effects of ginger, there are clinical trials that take advantage of the anti-inflammatory effects of ginger compared to conventional NSAIDs, such as ibuprofen [30,31]. In other clinical studies, ginger was shown to reduce knee pain significantly, either alone [32] or together with *Echinacea* extract (15 subjects > 60 years of age, 25 mg of ginger and 5 mg of *Echinaceae* extracts for 30 days) [33]. The studies of Haghighi and co-investigators [32] performed on 120 outpatients with osteoarthritis who were treated with 30 mg of ginger extract daily for one month showed improvement in the treated group that was similar to the results obtained for the group who received 400 mg ibuprofen administration. Some other trials found that it decreased pain and swelling in patients with osteoarthritis and rheumatoid arthritis [34]. Recent clinical research found that ginger was almost as effective as ibuprofen in relieving postoperative sequelae. This conclusion was drawn after the completion of a double-blind randomized clinical trial performed with the participation of 60 healthy adults [35]. Other clinical studies revealed that ginger acted effectively and similarly to Novafen in relieving primary dysmenorrhea. There were no statistical differences in the pain-killing action of ginger (200 mg of powder/ capsule) compared to the conventional NSAIDs in the clinical study of Rad and co-investigators, who obtained 168 questionnaires from girls in their early 20s. Therapy with ginger does not cause side effects. Thus, it can be used by the elderly and by patients with kidney and liver diseases or asthmatics [36,37].

Moreover, ginger, due to its anti-inflammatory properties, is considered as an appropriate drug for the treatment of migraine attacks [38] and as an analgesic and ergogenic agent in sport [39]. The individual compounds isolated from ginger have also been a topic of research with respect to their anti-inflammatory potential. 6-Shogaol, the phenolic component of ginger extract, in the studies of Chen and co-investigators was found to improve Nrf2 translocation and the heme oxidase-1 (HO-1) levels at the dose of 20 µM [40]. Nuclear factor erythroid 2-related factor (Nrf2) is responsible for normalizing the appearance of molecules working against oxidative stress [41]. Moreover, 6-gingerol, another phenolic component of ginger, decreased lipid peroxidation and increased the antioxidant activity of SOD, glutathione and peroxidase, suppressing oxidative stress [21]. Additionally, it was proven that 6-gingerol attenuated the synthesis of pro-inflammatory cytokines via the downregulation of p38 MAP kinase activity and NF-kB expression [42].

### 3.2. Influence of Ginger on the Cardiovascular System

In a clinical study based on a group of patients below 50 years of age, ginger was found to lower both systolic and diastolic blood pressure during ≤8 weeks of treatment with a ginger dose of ≥3 g per day. Its mechanism of action in this aspect has not yet been explored, but it is believed that its therapeutical potential may be connected to its antioxidant activity [43,44,45] due to the presence of phenolic compounds such as shogaols, zingerone, paradol and gingerols, which have been proven to be strong antioxidants [1]. The literature provides examples of the correlation between the antioxidant potential and functioning of the cardiovascular system. The regulation can be performed by reducing lipid peroxidation, which can cause vasoconstriction and blood pressure (BP) rise [46]. Additionally, these compounds are good vasodilators due to their action of increasing the level of plasma nitric oxide, which can also reduce the proinflammatory cytokine concentration and platelet aggregation [47].

Other studies demonstrated that ginger intake at different doses (0–2, 2–4 and 4–6 g/day) is responsible for lowering the spread of hypertension. The risk of hypertension decreased by 8% and 13% depending on the amount of ginger in the daily food ration [48]. There are also other clinical trials which tested the influence of ginger on the blood pressure. The study of Nayebifar and co-investigators, who tested the influence of ginger consumption (3 g a day for 10 weeks) on the blood pressure of overweight women undertaking intensive training, showed a significant decrease in the systolic pressure values. The administration of ginger with no training led to a decrease in the diastolic pressure as well [49].

In the published studies, 6-shoagol, a major constituent of ginger extract, was found to eliminate sleep apnea and relax the blood vessels in a guinea pig model. As a result, the animals were found to have decreased blood pressure. Additionally, the inotropic effect of the aqueous extract on the isolated left atrium of the rodents’ hearts was described [50].

Hypercholesterolemia is one of the key factors related to the circulation problems. Disturbed levels of lipoproteins, such as LDL-C and HDL-C, and triacylglycerols (TAG), called dyslipidemia, are a risk factor for cardiovascular disease (CHD) [51]. Makan Pourmasoumi and other researchers showed that high doses of ginger rhizomes (>2 g/day) lower the levels of TAG and LDL-C in the blood. Other studies confirmed these observations. According to Khosravani and co-investigators, treating a group of rats with ginger water extract (250 mg/kg b.w.) three times a week for 4 weeks, in combination with aerobic training, caused a reduction in the TAG, total cholesterol (TC) and LDL and a rise in the HDL levels in the blood [52]. Additionally, it was found that ginger ethanolic extract (200 mg/kg b.w.) lowered the total cholesterol levels in rabbits with streptozotocin-induced diabetes after 20 days of treatment [11], and in rats, an increased release of tissue biomarkers (LDH and AST) and increased cardiac biomarker CK-MB, cTn-I and cTN-T levels in the plasma were observed [53]. Moreover, ginger is responsible for an increase in cholesterol 7-alpha- hydroxylase activity, which participates in the removal of cholesterol from the body. In the study of Bhandari et al., ethanolic extract from ginger rhizomes was found to regulate the lipid levels in diabetic rats that were fed with 200 mg/kg b.w. of extract for 20 days [11]. Other reports demonstrated that the activity of the LDL receptors that remove cholesterol from the plasma rises after ginger treatment in rats with diabetes [54].

**Table 1:** Pharmacological effects of ginger supplementation in in vivo studies (↓—downregulation/decrease, ↑—upregulation/increase).

Extract/Natural Product	Study Group and Duration of Treatment	Dosage	Comments	References
**Ginger rhizome**	Patients ≤ 50 years 8 weeks	≥3 g per day	↓ Systolic and diastolic blood pressure, possibly due to antioxidant potential	[43,44]
**Ginger rhizome**	Patients	(0–2, 2–4 and 4–6 g/day)	↓Hypertension by 8 and 13%	[48,49]
**Ginger rhizome**	Patients	≥2 g per day	↓ TAG and LDL-C in blood	[51]
**Ginger extract**	120 patients	30 mg of ginger extract in 2 capsules a day for 1 month	↓ Pain ↑ Joint motion	[32]
**Ginger extract with Echinacea extract**	15 patients with osteoarthritis; >60 years of age; men/women ratio: 2/13	25 mg ginger and 5 mg of Echinacea extract, 30 days	↓ Pain ↓ Knee circumference	[33]
**Ginger rhizome**	60 patients with postoperative sequelae; men/women ratio: 24/36	500 mg of powdered ginger rhizome; oral administration, every 6 h after surgery	↓ Pain Similar effects to ibuprofen	
**Ginger rhizome**	24 healthy overweight women 20–30 years of age	3 g of powdered ginger a day; oral administration for 10 weeks with and without training	↓ Systolic blood pressure ↓ Diastolic blood pressure in the group treated with ginger with no training	[49]
**Ginger extract**	32 50-day-old male Sprague-Dawley rats	250 mg/kg b.w. water extract, 3 times a week; oral gavage, 4 weeks	↓TAG, TC and LDL; ↑HDL in combination with aerobic training ↑ LHD and AST biomarkers ↑ CK-MB, cTn-I and cTN-T in plasma	[52]
**Ginger extract**	24 male albino Wistar rats (200–250 g)	250 mg/kg b.w. of methanolic ginger extract daily for 3 weeks	↓Plasma sugar levels ↓ Hyperglycemia ↓ Hyperinsulinemia	[55]
**Ginger juice**	Rats with alloxan-induced diabetes (150 mg/kg b.w.)	4 mL/kg b.w. daily, oral administration for 6 weeks	↓Plasma sugar levels ↑ HDL ↓ TC, TAG, LDL, creatinine, uric acid	[54]
**Ginger extract**	Rats with streptozotocin-induced diabetes	200 mg/kg b.w. of water extract, 20 days	↓ Hyperglycemia ↓ TC ↑ Cholesterol 7-alpha-hydroxylase activity ↓ Levels of liver and pancreas thiobarbituric-acid-reactive substances	[11]
**6-Shogaol**	Guinea pig	-	↓ Sleep apnea ↑ Relaxation of blood vessels ↓ Blood pressure	[50]
**Ginger extract**	7-week-old male mice [55] and male Sprague-Dawley rats [55]	0.3–0.4% ginger extract added to the high-sugar and high-fat diet feed for 2 weeks [55] and 35 days [55]	↓ Obesity by PPAR receptor regulation ↓ Body weight ↑Energy expenditure, heat production ↓ Hyperglycemia	[55,56]
**Ginger extract**	24 male Sprague-Dawley rats	20 and 50 mg/kg b.w. of 95% ethanol extract daily by oral gavage for 5 weeks together with fructose solution	↑ Heat production ↓Fructose-overconsumption-induced adipose tissue insulin resistance in rats ↓ Cytokines	[57]
**Ginger essential oils**	Rats	12 weeks	Treatment of nonalcoholic fatty liver disease → Modulation of the hepatic-SREBP-1c- and CYP2E1-mediated pathway ↓ Hyperlipidemia ↓TG, TC	[58]
**Ginger extract**	40 5-week-old female BALB/c mice with induced colitis	p.o., 100, 300 and 500 mg/kg b.w. 21 days, once a day	↓ Body weight ↓ Colon length ↓ Number of neutrophils and pro-inflammatory cytokines (IL-6 and TNF-α)	[22]
**6-Gingerol**	40 I/R-induced male Sprague-Dawley rats	Oral gavage, 25 and 50 mg/kg b.w., 3 days before operation, once a day	↓ IL-1β, IL-6 and TNF-α	[21]
**6-Shogaol**			Improved translocation of Nrf2 factor ↑ OH-1	[40]
**6-Gingerol**	40 I/R-induced Male Sprague-Dawley rats	Oral gavage, 25 and 50 mg/kg b.w., 3 days before operation, once a day	↓ Lipid peroxidation ↑ SOD, glutathione and peroxidase activity	[21]
**Ginger extract with *Lactobacillus acidophilus***	75 Wistar rats, 8–10 weeks old with DMH-induced inflammation	50 mg/kg of ginger CO_2_ extract and 10^9^ CFU of *L. acidophilus*; 1 month, once a day	↓ MDA, TNF-α, COX-2, iNOS, c-Myc ↑ CAT, SOD levels	[23]
**GEO**	Mice with autoimmune encephalomyelitis (EAE)	200 and 300 mg/kg	↓ IL-12, TGF-β	[24]
**GEO**	48 male albino rats, 12 weeks old, with induced nephrotoxicity	Essential oil from ginger, 50 mg/kg b.w.; oral administration	↓ Urea, creatinine and blood urea nitrogen ↑ Renoprotection ↑ Adenosine deaminase activity resulting in neuroprotective effect	[25,26]
**6-Gingerol**	Streptozotocin-induced (50mg/kg) 7-week-old male Wistar rats	75 mg/kg/day oral administration	↓ Acetylcholinesterase levels ↓ Pro-inflammatory cytokines Treatment of Alzheimer’s disease ↓ BWG%, ↓ GSH/GSSG	[59]

The data described above contribute to the statement that ginger can be used as a cardioprotective agent that acts beneficially through the protection of cardiovascular system.

### 3.3. Ginger as an Anti-Obesity and Anti-Diabetes Mellitus Drug

Ginger extract is also taken into account as a possible anti-obesity drug. It was proven that the administration of 50% ethanolic extract from ginger rhizomes may prevent the development of obesity [55] and insulin resistance in rats [55] inter alia by regulating the PPAR receptors. Properties similar to those of the total extract were assessed in the ginger constituents 6-gingerol and 6-shogaol [55,56]. Other studies revealed that 95% ginger ethanolic extract administered to male Sprague-Dawley rats increased the energy expenditure of the body, decreased the size of the adipocytes and led to the activation of the thermoregulation system in the adipose tissue by increasing the level of heat production. The authors concluded that supplementation with ginger extracts together with a high-fructose diet, leads to a reduction in the insulin resistance effects and suppresses the activity of proinflammatory cytokines that are related to the adipose tissue macrophages [57]. Moreover, the latest research proved that ginger administered to male rats improved the composition of the gut microbiota, which was destroyed by a high-fat diet, which may be important for restoring the organism’s equilibrium (HFD) [57,60].

Obesity is closely related to the development of diabetes [61]. Diabetes mellitus is defined as a metabolic disorder indirectly caused by changes in lifestyle and living environment, and it affects humans all around the world at an alarming rate. Data provided by the International Diabetes Federation (IDF) show that in 2019, over 460 million adults in the age range of 20–79 were living with this disease. It is predicted that in 2045, this number will exceed 700 million. Diabetes occurs when the pancreas can no longer create insulin or when the body is not able to make proper use of it. Therefore, patients with diabetes share common characteristics, mainly raised blood sugar levels, decreased insulin sensitivity, obesity, dyslipidemia and hypertension, which almost always appear simultaneously. If left untreated or treated inappropriately, the disease will harm the entire body. Its broad complications are distinguished as neurological, cardiological, nephrological, ophthalmic, sexual, dental, immunological and skin disorders, as well as others. Poor outcomes and prognoses are related to the microvascular changes that result from the activation of the metabolic and biochemical pathways as a result of diabetes.

From the molecular point of view, the activation of the polyol and hexosamine pathways, the activation of protein kinase C and the increase in the amount of glycation end products in the blood are described in the pathogenesis of diabetes. Oxidative stress and inflammation, which lead to functional and structural abnormalities, are typical of the progression of the disease, as they lead to vascular wall integrity attenuation and, furthermore, to increased permeability and local ischemia [46,47,62]. The effectiveness of ginger-based therapies for type 2 diabetes mellitus (T2DM) and metabolic syndrome may be due to the presence of polyphenols in ginger extracts. The administration of ginger extracts or single components improves the leptin levels in diabetic patients and can be used to treat disorders caused by diabetes [57]. In the study of Kadnur and Goyal [63], 3-week-long oral supplementation with the methanolic extract of dried ginger rhizomes (250 mg/kg b.w.) led to reduced fructose-induced disturbance of the plasma sugar and lipid levels and regulated hyperinsulinemia in male albino Wistar rats. According to the authors, these properties were correlated with the presence of 6-gingerol in the tested sample. Another study performed on rats with streptozocin-induced diabetes that were supplemented orally with 90% ethanolic extract from ginger rhizomes (200 mg/kg b.w.) showed lower levels of liver and pancreas thiobarbituric-acid-reactive substances in the animals, similar to the registered drug, gliclazide (25 mg/kg b.w., p.o.) [11]. The serum parameters of diabetic rats were also tested by Elshater and co-investigators. In the alloxan model of diabetes, the oral administration of ginger juice squeezed from the fresh rhizome (4 mL/kg b.w.) to both the pre-treated and post-treated animals for six weeks showed a marked reduction in the plasma glucose levels. Decreased levels of TC, TAG, LDL, creatinine and uric acid in the post-treated animals were also observed, together with an increased level of HDL cholesterol [54]. The association between diabetes and hypercholesterolemia is broadly discussed, because atherosclerosis after dyslipidemia is the most prevalent complication of diabetes mellitus. This is why the introduction of ginger into daily food rations is of great importance [64,65]. A previously published meta-analysis showed that ginger was able to significantly lower the FBG (fasting blood glucose) and HbA1c (glycosylated hemoglobin) levels and improve the pancreatic function and insulin resistance indices, namely INS (fasting insulin) and HOMA-IR (homeostasis model assessment–insulin resistance index). In addition, most of the MetS risk factors were mitigated by ginger preparations [4].

### 3.4. The Treatment of Nonalcoholic Fatty Liver Disease

Another disease related to obesity, dyslipidemia, hypertension and type 2 diabetes mellitus is nonalcoholic fatty liver disease (NAFLD), in which the lipid metabolism is disturbed. Free fatty acids released into the circulation system stimulate the synthesis of HMGCR, SREBP1, ACC and FAS. These enzymes cause the synthesis of liver lipids, which leads to hepatic steatosis followed by hepatic lipid accumulation and, finally, NAFLD. Additionally, the presence of FFA stimulates the expression of the cytochrome P450 isoform 2E1 and production of ROS. Therefore, increased liver inflammation and oxidative stress lead to irreversible changes in the liver tissue [58]. Based on recent studies, it was found that supplementation with ginger essential oil (GEO) for 12 weeks caused decreased TG and TC levels in the serum of high-fat-diet-induced rats. Next, GEO led to the reduction in hyperlipidemia and obesity, connected with an increased mass of adipose tissue. It is understood that GEO, due to its antioxidant and anti-inflammatory properties, acts on the biosynthesis of fatty acids and cholesterol through the modulation of the hepatic SREBP-1c- and CYP2E1-mediated pathway [66].

### 3.5. Application of Ginger to Treat Alcohol Addiction

Long-term alcohol exposure can lead to histopathologic changes in different organs [67]. In the lungs, there are alterations such as the enlargement and destruction of the air spaces, focal infiltration of the polymorphonuclear cells, an increase in the pneumocytes in the alveolar walls and fibrosis with damage to the lung structure, which was observed during experiments on rats. Moreover, recent studies revealed a rise in 8-hydroxy-2′-deoxyguanosine (8-OHdG), NADPH oxidase and Ox-LDL in the lung tissue after ethanol exposure. Alterations in these parameters mean that ethanol exerts its effects via oxidative stress. The studies showed that ginger supplementation leads to an increase in the antioxidant parameters, decrease in lipid and protein oxidation and a reduction in the levels of pro-inflammatory cytokines (TNF-α and IL-6). Ginger was stated to inhibit prostaglandin and leukotriene synthesis via the dampening of 5-lipooxygenase synthetase activity [68]. The other studies found that ginger had anti-fibrotic properties, because it was responsible for decreasing the number of PCNA-positive cells in the lungs [69].

### 3.6. Ginger in the Treatment of Prostate Complications

Due to the broad spectrum of properties of ginger, namely its antihyperglycemic, anti-inflammatory, antiangiogenic and cytotoxic effects on cancer cells, ginger can be used for the prevention and treatment of prostate complications, which are diagnosed in diabetic patients.

Microvascular complications leading to prostate disorders are a result of oxidative stress and diabetes mellitus, and this, in turn, leads to the inflammation of the prostate. The literature reveals that ginger and its active constituents, namely 6-gingerol, zingerone and geraniol, have anti-inflammatory and androgenic activities, which suggests that these compounds have a protective effect on the prostate gland [59]. It was reported that ginger has positive effects on sperm viability and mobility, which explains why the administration of ginger is used to maintain male reproductive functions [59]. Another study showed that treatment with geraniol and 6-gingerol has positive effects on the body weight and key oxidative stress markers. These compounds decrease the levels of malondialdehyde significantly, and the body weight gain percentage (BWG%) was significantly lower in comparison to diabetic rats, respectively. It was proved that administration of 6-gingerol caused a reduction in inflammatory activities and improved the histopathological profile [59].

## 4. Conclusions

Ginger is certainly one of the most commonly used plant medicines in the world. Numerous in vitro studies, together with more frequent in vivo research on animals and humans, provide many conclusions about the therapeutic potential of this plant. It is very important to note that the directions of scientific research increasingly tend to study the activity of single compounds isolated from ginger. The multitude of literature data presented in this work allows us to state that ginger rhizomes have the ability to inhibit the response of the innate immunological system. They are also characterized by antioxidant and anti-diabetic properties. They reduce the levels of LDL cholesterol and triglycerides in the blood and also increase the concentration of HDL cholesterol. In light of the scientific data, it can be concluded that ginger supplementation has supportive effects on the treatment of inflammatory disorders and metabolic syndrome.

## Data Availability

Not applicable.

## Abbreviations

AD	Alzheimer’s disease
ADA	Adenosine deaminase activity
BUN	Blood urea nitrogen
BWG%	Body weight gain percent
c-Myc	Multifunctional transcription factor
CAT	Catalase
COX-2	Cyclooxygenase 2
DAI	Disease activity index
EAE	Experimental autoimmune encephalomyelitis
ERK1/2	MAPK3 mitogen-activated protein kinase 3
FAS	Fetal alcohol syndrome
FBG	Fasting blood glucose
GSH	Glutathione
GSSG	Oxidized glutathione
HDL	High-density lipoprotein
HDL-C	High-density lipoprotein cholesterol
HMGCR	3-Hydroxy-3-methylglutaryl-CoA reductase
HO-1	Heme oxygenase-1
IC50	Half-maximal inhibitory concentration
IL-8	Interleukin-8
IL-1 α	Interleukin 1-α
IL-6	Interleukin 6
IL-1β	Interleukin 1β
IL-10	Interleukin 10
iNOS	Inducible nitric oxide synthase
5-LOX	5-Lipooxygenase
LDL-C	Low-density lipoprotein cholesterol
MAPK	Mitogen-activated protein kinases
MDA	Malondialdehyde
MPO	Myeloperoxidase
mTOR	The mammalian target of rapamycin
NADP	Nicotinamide adenine dinucleotide phosphate
NAFLD	Nonalcoholic fatty liver disease
NF-κB	Nuclear factor kappa-light-chain-enhancer of activated B cells
Nrf2	Nrf2 transcription factor
NSAIDs	Non-steroidal anti-inflammatory drugs
8-OHdG	Deoxyguanosine
Ox-LDL	Oxidized low-density lipoprotein
PCNA	Proliferating cell nuclear antigen
PGE2	Prostaglandin E2
PPAR	Peroxisome proliferator-activated receptor
ROS	Reactive oxygen species
SOD	Superoxide dismutase
SREBP1	Sterol regulatory element-binding protein 1
T2DM	Type 2 diabetes mellitus
TAG	Triacylglycerols
TC	Total cholesterol
TGF-β	Transforming growth factor β
TNFα	Tumor necrosis factor alpha
ZO-1	Zonula occludens-1 protein
